# Economically optimal timing for crop disease control under uncertainty: an options approach

**DOI:** 10.1098/rsif.2010.0056

**Published:** 2010-04-07

**Authors:** Martial L. Ndeffo Mbah, Graeme A. Forster, Justus H. Wesseler, Christopher A. Gilligan

**Affiliations:** 1 Department of Plant Sciences, University of Cambridge, Downing Street, Cambridge CB2 3EA, UK; 2 Environmental Economics Group, Social Science Department, Wageningen University, The Netherlands

**Keywords:** disease control, stochastic epidemics, density dependence, optimal timing, real options

## Abstract

Severe large-scale disease and pest infestations in agricultural regions can cause significant economic damage. Understanding if and when disease control measures should be taken in the presence of risk and uncertainty is a key issue. We develop a framework to examine the economically optimal timing of treatment. The decision to treat should only be undertaken when the benefits exceed the costs by a certain amount and not if they are merely equal to or greater than the costs as standard net-present-value (NPV) analysis suggests. This criterion leads to a reduction in fungicide use. We investigate the effect of the model for disease progress on the value required for immediate treatment by comparing two standard models for disease increase (exponential and logistic growth). Analyses show that the threshold value of benefits required for immediate release of treatment varies significantly with the relative duration of the agricultural season, the intrinsic rate of increase of the disease and the level of uncertainty in disease progression. In comparing the performance of the delay strategy introduced here with the conventional NPV approach, we show how the degree of uncertainty affects the benefits of delaying control.

## 1. Introduction

Diseases of agricultural crops continue to cause severe losses and to pose a threat to food security and to the sustainability of crop production across large regions of the world (Strange & Scott 2005; Oerke 2006; Gilligan 2008). Some of these threats involve emerging or re-emerging pathogens, exemplified by new strains of cassava mosaic virus in Africa (Strange & Scott 2005) and of wheat stem rust (Strange & Scott 2005; Wanyera *et al.* 2006) in Africa, Asia and the Middle East. The risks, however, of severe enough outbreaks to merit control over large regions often vary from year to year. Such variability reflects the inherent stochasticity of epidemics as well as uncertainties associated with weather patterns. For many crop diseases, chemical control remains the principal means of disease reduction and eradication (Cook *et al.* 1999; Paveley *et al.* 2008). Chemical control encompasses a wide range of pesticides and fungicides. Whereas, routine application of chemical control may once have been possible when labour and materials were comparatively cheap, increasing costs together with concerns about hazards to non-target organisms and the risks of selecting for fungicide resistance in target populations militate against routine use in many crops (Waard *et al.* 1993). How and when, if at all, to deploy expensive methods for disease control over large regions in the face of uncertainty remains a major scientific challenge. The problem is important for individual farmers and for advisory and regulatory agencies that seek to optimize the deployment of disease control over large areas. Here, we focus on the problem from the perspective of a regulator charged with optimizing the benefit to a population of growers. The challenge lies in combining epidemiological models for disease outbreaks with economic models in the presence of uncertainty, in order to identify the optimal timing to apply control. Clearly, failure to control early enough when an outbreak occurs can lead to severe crop loss. Unnecessary deployment also incurs direct losses through costs for fungicides, pesticides and additional labour; it may also lead to indirect costs when over-use of a chemical results in selecting for resistant strains in the target population.

In this paper, we focus on how to optimize the timing of a single application of a treatment to a regional agricultural monoculture or perennial crop, over which there is a risk of severe disease. We motivate the analyses for systems in which a single application of a fungicide can be used to control diseases such as rusts, and certain mildews and blights. Typical examples include, but are not limited to, *Phakospora pachyrizi* and *Microsphaera diffusa* on soybean crops and *Rhizoctonia solani*, which causes sheath blight on rice. We consider fungicidal treatments with protectant and/or eradicant action (Cook *et al.* 1999; Leyra *et al.* 2008; Paveley *et al.* 2008) that differ in their epidemiological modes of action. Protectant fungicides, typified by systemic activity, act to protect plants from subsequent infection, while eradicants, typified by contact fungicides, eliminate existing infections. Typical examples of fungicides with dual protectant and eradicant activity are azoxystobin for control of sheath blight on rice (Groth 2008), and tebuconazole for control of powdery mildew (*M. diffusa*) on soybean (Yorinori *et al.* 2004).

In order to couple epidemiological uncertainty (Gubbins *et al.* 2000; Gilligan 2002) with economic analysis, we adopt an options approach (Saphores 2000; Gilligan 2003; Matty *et al.* 2004). By evaluating treatment as an option which can be exercised during the season, we go a step further than the standard cost–benefit analysis, which identifies the optimal treatment time as the point at which the benefits of treatment are merely equal to or greater than the costs. We show that, if the threshold levels of benefits over costs have been met, meaning the expected gain from immediate treatment is positive, there is still value in waiting. This supports the conclusions of Kuosmanen *et al.* (2006) and Lichtenberg & Zilberman (1986), who advocate a reduction in the use of pesticides in the form of a delay to treatment. The delay hinges upon key features of the system, namely uncertainty in disease progression, irreversibility of the decision to treat, and flexibility to delay treatment.

The convention in options analysis of biological systems is to assume exponential increase in the state variable (i.e. the number of diseased hosts). In practice most systems exhibit some form of density dependence, even early in the spread of disease, as the availability of susceptible host tissue becomes limiting (Burdon & Chilvers 1982). Accordingly, we introduce a simple form of density dependence in disease increase (modelled by logistic growth) and show how this can profoundly affect the conclusions about when to treat compared with the conventional assumption of exponential increase in disease.

The delay strategy based upon the real options approach maximizes the expected gain in the presence of uncertainty. We examine the effect of applying the delay strategy to epidemics with different degrees of uncertainty, as well as the impact of density-dependent compared with density-independent growth on decision-making. Finally, we compare the standard, cost–benefit approach to the real options approach for decision-making, and discuss the relevance of our results to disease control policies.

## 2. Model

Consider agricultural disease outbreaks in a particular crop at the landscape scale, and a decision-making agency with the authority to decide whether or not treatment should be administered. Given a suitable (i.e. biologically validated) model for disease spread, the decision-making agency requires (i) knowledge of the current status of disease and (ii) estimates of the model parameters in order to predict future spread of disease. Here, we consider two classes of parameters; one is concerned with the expected rate of spread of disease, the other is a measure of the within-season variability in the disease dynamics. The latter is also known as the volatility of disease increase (Gilligan 2003). Estimates of the parameters may be derived from the current epidemic, for example using conditional least squares or maximum likelihood methods (Marcus 1991; Forsyth 2000). Exceptionally, for some recurrent epidemics, estimates of the parameters may be known from previous disease outbreaks (Kleczkowski & Gilligan 2007). Knowing the current and probable future state of the epidemic, the decision-making agency can either approve or postpone the decision to deploy disease control. We assume that the objective for the agency is to maximize the expected return for that season in the presence of uncertainty. The seasonal return depends upon the quantity of healthy yield, the market price of the crop and the cost of spraying the region with fungicide. The degree of uncertainty over the market price of the crop at the end of the season and the cost of spraying the region with fungicides can be minimized by entering a contract at the beginning of the season, to sell at an agreed price at harvest. This type of arrangement, known as a forward contract, provides the seller with insurance from the purchaser in the form of a margin account (Hull 2002). Hence, we assume that the market price and treatment cost are easily quantifiable. Therefore, the only major concern for the grower would be the quantity of healthy yield, which we assume is directly related to the level of disease present during the season.

Disease spread is highly stochastic. The stochasticity derives not only from environmental fluctuations, such as weather variability and soil quality, but also from demographic uncertainty, associated with the transmission of infection. Many experimental data show that the cumulative number of infected sites typically follows a logistic growth curve, equating to rapid increase in infections when susceptible sites are abundant and a deceleration of the epidemic as susceptible sites become scarce. In larger systems, where the number of susceptibles is very large during the time scale of control, density dependence is less evident. We assume that disease is spread from sources of infection to neighbouring fields by movement of pathogen propagules, such as spores. Subsequent amplification within an infested field provides a further source for pathogen spread. The unit of infection (*I*(*t*)) may vary from a single plant to an entire field, depending upon the scale of interest (Gilligan 2008).

The expected return for the season is related to the treatment costs (denoted by *D*) and the quantity of healthy yield, which in turn is related to the severity of disease and the action of the fungicide. Once fungicides have been applied the costs of treatment (*D*) cannot be reversed. The treatment costs (*D*) are therefore considered to be irreversible. To quantify the positive effect of treatment on yield, we use a value function (*V* = *V*(*I, t*)). The value of treatment is the product of the yield gained from releasing the fungicide at time *t* when the level of infection in the system is *I*(*t*), multiplied by the unit price of the commodity. The value of treatment is then related to the amount of infection prevented. The objective is to maximize the expected value of treatment (the expected value is a measure of the expected yield under uncertainty) minus the cost of treatment (*D*). The flexibility in choosing the timing of treatment in combination with uncertainty about the value of treatment (*V*) and irreversibility of treatment costs (*D*) creates a value known as the real option value in the finance literature (Wesseler 2003). The value of the option to treat depends on the current value of treatment as well as the value of treatment at a later time. It is always greater than or equal to the current value of treatment (Hull 2002). We denote the value of the option to treat by *F*(*V, t*_0_), and we require a rule that maximizes this value. Since the net gain from treatment is the difference between the value of treatment at future time t_*_ and the treatment costs, *V*(I, t_*_) − *D*, we wish to maximize the value of the option to treat, that is
2.1
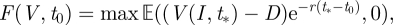

where 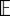 denotes the expected value of the net benefit of treatment under uncertainty and *r* is a discount rate that is conventionally used in economic models, whereby expenditure today has greater weighting than the same expenditure at some time in the future. Note the value of the option to treat is uncertain and is influenced by the point in time in the future when treatment is exercised. We consider two standard functions, the exponential and the logistic, to model the trend in disease increase. Hence for density-independent growth the level of infection is modelled by a simple geometric Brownian motion, given by
2.2
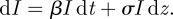

For density-dependent growth, the increase in infection is modelled using the following stochastic logistic differential equation:
2.3
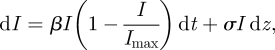

where *β* is the transmission rate of the pathogen, *σ* is the level of uncertainty in the path of infection, *I*_max_ is the maximum amount of infection relative to the environmental carrying capacity (Marcus 1991), and d*z* is a Gaussian distributed Wiener process, with zero mean and infinitesimal variance d*t*, which is used to model the background fluctuations in disease severity (see the electronic supplementary material). The model in equation (2.3) is analogous to an SI (susceptible-infected) epidemiological model.

## 3. Methods

The objective of the decision-making agency is to maximize the option to treat that is given by equation (2.1), reproduced here for convenience:
3.1
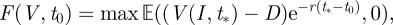

where 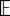 denotes the expectation and *r* is a discount rate. For every model of disease increase (exponential and logistic increase), the value of treatment, *V*(*I, t*), is given by
3.2
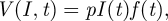

where *p* is the monetary gain in yield per unit of infection prevented by treatment, *I* is the level of infection at time *t* and *f*(*t*) is the scaled value of the expected value of the amount of infection averted by treatment (see the electronic supplementary material). Since *I* follows the Ito process (Dixit & Pindyck 1994) given by equation (2.2) or equation (2.3), which incorporates both the demographic trend and uncertainty of the epidemic progression, *V*(*I, t*) follows the process:
3.3
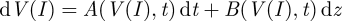

(see the electronic supplementary material for further details and the assumptions underlying the Ito process).

We formulate the decision problem of whether to treat or postpone the treatment as an optimal stopping problem (Wesseler 2003). Using standard methods from dynamic programming and Ito calculus (Dixit & Pindyck 1994), we show that solving the above problem (equation (3.1)) is equivalent to solving the following free boundary value problem (see the electronic supplementary material for details):
3.4
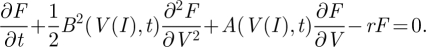

In addition, *F*(*V, t*) must satisfy the following boundary conditions:
3.5
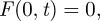

3.6
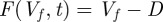

3.7
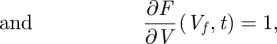

in which equations (3.5)–(3.6) follow naturally from the formulation of the problem while equation (3.7), known as the smooth-passing condition, is typical for optimal stopping problems (Dixit & Pindyck 1994). The principal parameters and variables used in the model are summarized in table 1.

## 4. Results

The results presented here give insight into optimal timing of treatment for disease control in an agricultural landscape during a single season. Given some prior knowledge of the epidemiological and economic parameters, we can calculate the value of treatment required for which immediate treatment is optimal. The decision to treat depends upon the progression of the epidemic, since we assume that the cost of treatment (*D*) is known. The value of treatment at any time is therefore estimated from the current level of infection (using equation (2.2) or equation (2.3)).

Using a successive over-relaxation algorithm (Wilmott *et al.* 1995), we solved the free boundary value problem (equations (3.4)–(3.7), §3) to obtain a value of the option to treat (*F*(*V, t*)) at different times and for different levels of infection. We also calculated the threshold value of treatment *V*_*f*_ at different times. At a given time *t*, treatment should be applied if the value of treatment *V* is greater than or equal to the threshold value *V*_*f*_ (see the electronic supplementary material). It follows that *V*_*f*_ is the value required for immediate treatment, which varies with the economic and epidemiological parameters appropriate to the particular host–pathogen system. We analysed the sensitivities of the responses to changes in epidemiological parameters (*β*, *σ*) for both density-independent (exponential) and density-dependent (logistic) increase in infection and disease. Here we summarize the principal effects.

**Figure 1.**
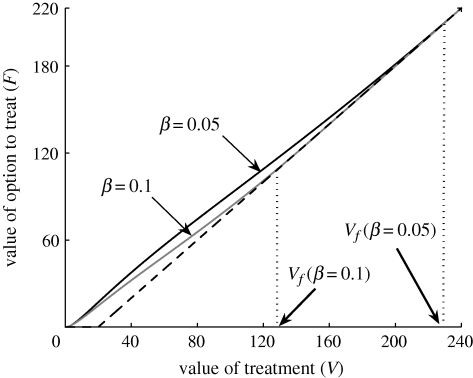
Relationship between the current value of the option to treat (*F*) and the value of treatment (*V*) for varying disease transmission rate (*β*) for logistic increase in disease. The dashed line represents the intrinsic value of the option (*V* − *D*) and the dotted lines are the projections from *F* (at *F* = *V* − *D*) on *V* to identify the threshold value of treatment (*V*_*f*_). *V*_*f*_(*β* = 0.1) and *V*_*f*_(*β* = 0.05) are the threshold values of treatment when the disease transmission rate (*β*) is equal to 0.1 (0.05). Default parameter values are *T* = 100 d (duration of the epidemic), *α* = 3 d (duration of protectant activity of treatment), *r* = 0.1 d^−1^ (discount rate), *I*_0_/*I*_max_ = 0.05 (initial proportion of infection relative to the environmental carrying capacity), *D* = 20 (costs of treatment), and *p* = 1 (monetary gain in yield per unit of averted infection). The relative magnitudes for costs of treatment and monetary gain from yield (through application of treatment) are expressed in arbitrary units.

Changing the transmission rate (*β*) has a marked effect on the threshold value required for immediate treatment (*V*_*f*_). The effect is especially pronounced for a logistic increase in infection (figure 1): where, if the spread of infection is rapid, treatment should be applied early. Moreover, there are substantial differences between the threshold values of treatment (*V*_*f*_(*β* = 0.05),*V*_*f*_(*β* = 0.1)) and the treatment value *V*, where the value of the option is said ‘to be in the money’ i.e. *V* >*D*. These differences indicate that consideration of uncertainty, irreversibility and flexibility generates huge additional value (figure 1). For a given value of the transmission rate, the threshold value of treatment differs if disease increase is modelled as a density-dependent function (e.g. using the logistic function) or as a density-independent function (using an exponential function). Our results show that it is not optimal to treat early in the epidemic (i.e. *t* < t*), when the increase in disease is assumed to be exponential (see the electronic supplementary material). However this is not the case with logistic increase, where it may be optimal to treat even at an early stage of an epidemic. It follows that selecting an appropriate and biologically plausible model for disease increase is important in devising effective models for disease control.

**Figure 2.**
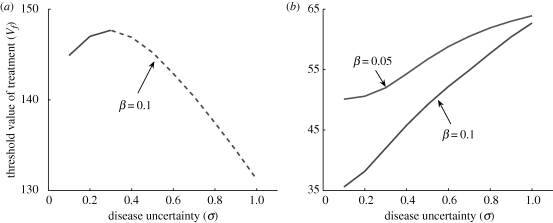
Relationship between the threshold value of treatment (*V*_*f*_) and the level of uncertainty in disease severity (*σ*), derived numerically. (*a*) *V*_*f*_ at initial time, for disease increase modelled by a logistic function and disease transmission rate (*β*) equal to 0.1. The solid line represents *increasing value* and the dashed line represents *decreasing value*. (*b*) *V*_*f*_ at a given time *t* > t* (see the electronic supplementary material), for an exponential model for disease increase and disease transmission rate (*β*), respectively, equal to 0.05 and 0.1. Default parameter values are given in figure 1.

Our results show a concave response in the threshold value of treatment (*V*_*f*_) as a function of the level of uncertainty in the path of infection (*σ*) when disease increase is logistic (figure 2*a*; see also the electronic supplementary material). The concavity infers that there is a critical value σ* such that for *σ* > σ* the severity of infection is not only highly variable and hence uncertain, but that the level of infection will almost surely decrease towards the end of the season, providing the season is long enough to allow extinction (see the electronic supplementary material). The value of σ* depends upon the value of the transmission rate (*β*). For relatively short seasons, *V*_*f*_ is an increasing function of the level of uncertainty (*σ*) for both exponential and logistic models for disease increase.

The results above show that, at any point in the season, there exists a level of infection above which it is optimal to treat. If the level of infection has not yet reached that threshold, it is then optimal to wait longer. The option is said to have a value of waiting (named the *time value* in the economic literature), which derives from the uncertainty in disease progress. The waiting time reflects the desire to acquire more information about disease progress before carrying out treatment.

**Figure 3.**
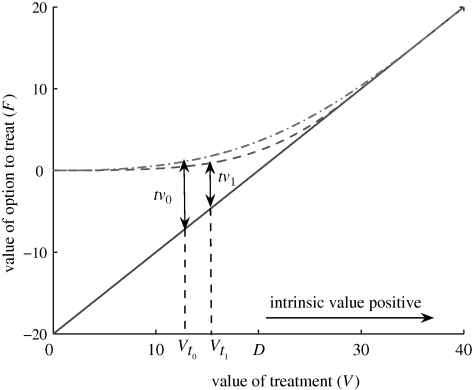
The value of treatment (*V*) and the value of the option (*F*) both change simultaneously with time. When the difference between the current value of the option (*F*) and its intrinsic value (*V* − *D*), known as the *time value* (*tv*), goes to zero the option should be exercised (i.e. the option is equal to its intrinsic value). The figure shows the derivation of the *time value* at two consecutive times, *t*_0_ and *t*_1_. It is assumed that at these times, the values of treatment are *V*_*t*_0__ and *V*_*t*_1__. Using these values and values of the option to treat, *F*(*V*_*t*_0__, *t*_0_) and *F*(*V*_*t*_1__, *t*_1_), we calculate the time values *tv*_0_ = *F*(*V*_*t*_0__, *t*_0_) − (*V*_*t*_0__ − *D*) and *tv*_1_ = *F*(*V*_*t*_1__, *t*_1_) − (*V*_*t*_1__ − *D*). The solid line denotes the intrinsic value of the option to treat (*V* − *D*), *F*_*t*_0__ (dot-dashed line) and *F*_*t*_1__ (dashed line) stand, respectively, for the values of the option to treat at *t* = *t*_0_ and *t* = *t*_1_.

Using the real options approach, we are able to estimate the time of release of treatment during the season that maximizes our expected gain in the presence of uncertainty about the future of disease progression. Since expectation is an average, it provides little information on the variability of the return among seasons or across independent sites. To demonstrate how this variability could play a role in policy decisions, we consider replicated epidemics with a range of values of the level of disease uncertainty *σ*. Each epidemic simulation produces a stochastically evolving value function (*V*(*I, t*), see the electronic supplementary material). Using the solution derived numerically for *F*(*V, t*) (from equation (3.7)), we obtain a corresponding value for the option to treat. From this we derive a time-value, which is the value of the option minus the intrinsic value of the option to treat (figure 3). The point at which the time-value drops to zero is the time at which treatment is optimal.

To illustrate results on the optimal time to release treatment, we first consider the effect of the level of disease uncertainty (*σ*) on the optimal treatment time conditional upon release of treatment (see the electronic supplementary material, figure S2). Treatment is released only if the *V*_*f*_ criterion is satisfied. This happens at time *t* if the value of treatment (*V*(*t*)) is greater than or equal to the threshold value of treatment (*V*_*f*_(*t*)). On average, the optimal time to treat is a decreasing function of the level of disease uncertainty but the variability changes with the model for disease increase (logistic or exponential growth). For logistic increase, the variability in the optimal time of treatment is a concave function of disease uncertainty (see the electronic supplementary material, figure S2*a*). In fact, for low values of *σ* (more predictable epidemics) the variability of the value of the time of treatment is an increasing function of disease uncertainty. Whereas for high values of *σ* (more unpredictable epidemics), variability of treatment time is a decreasing function of disease uncertainty. For exponential increase, the variability of the value of the time of treatment is shown to be a decreasing function of disease uncertainty (see the electronic supplementary material, figure S2*b*). Next we analyse the effect of the level of disease uncertainty on the probability of exercising the option to treat (see the electronic supplementary material, figure S3*a*,*b*). For convenience, we show the probability that the condition is not met. The more unpredictable an epidemic is, the less likely it is to reach the level of infection required for optimal release of treatment (see the electronic supplementary material, figure S3*a*,*b*).

We compare the real option approach with an alternative decision criterion, the standard cost–benefit approach in which treatment can be released as soon as the value of treatment is greater than the cost. Unsurprisingly, the divergence in the probability of treating on the real option over the standard cost–benefit approach increases with the level of disease uncertainty *σ* (see the electronic supplementary material, figure S3*c*,*d*). The effect is more pronounced for epidemics with density-independent than density-dependent growth rates when *σ* is large.

The gains from waiting for the standard cost–benefit and real option approach are defined, respectively, as
4.1
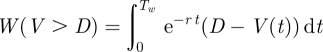

and
4.2
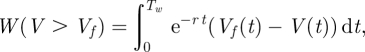

where *T*_*w*_ is the first time when either (*V*(*t*) > *D*) or (*V*(*t*) > *V*_*f*_ (*t*)).

For any given disease outbreak, one of the following three scenarios will be observed:

1. *V* < *D* for the entire season.
2. *V* ≥ *D* at some point during the season but *V* < *V*_*f*_ for the entire season.
3. *V* ≥ *V*_*f*_ at some point during the season.

**Figure 4.**
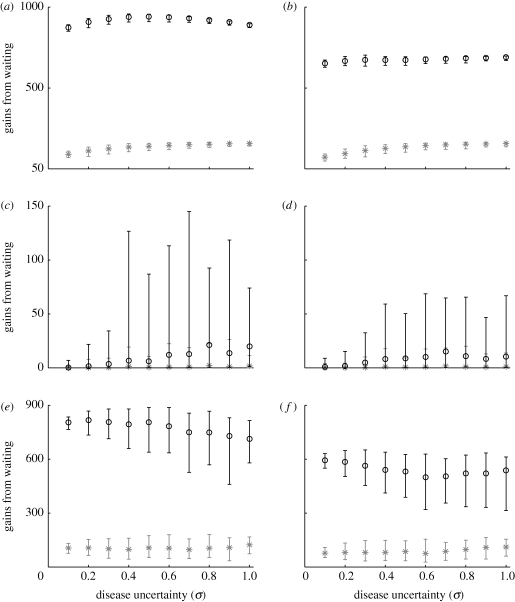
Gains from waiting under the standard (asterisks) and the real option (open circles) decision approaches for different values of the level of uncertainty in disease severity (*σ*), under an assumption of logistic (*a*,*c*,*e*) and exponential (*b*,*d*,*f*) increase in disease. Gains are shown, respectively, for the three possible outcomes (scenarios) of the treatment value. First scenario (*a*,*b*): *V* < *D* for the entire season. Second scenario (*c*,*d*): *V* > *D* at some point during the season but *V* < *V*_*f*_ throughout the entire season. Third scenario (*e*,*f*): *V* > *V*_*f*_ at some point during the season. The edges of the error bars show, respectively, the fifth and ninety-fifth percentiles, whereas the circle and the star denote, respectively, the mean value of the gains from waiting under the real option approach and the standard approach. In (*c*,*d*), the gains under the standard approach represent the economic gains obtained when *V* > *D*.

The gains from waiting reflect the gains from delaying treatment, in which the decision-maker balances the short-term loss with an expected long-term gain. The differences between average gains from waiting under the real option approach and the standard cost–benefit approach are larger for the logistic model than for the exponential model (see figure 4). If disease increase is a density-independent (exponential) process, a decision-making agency would have more incentive to aim for the long-term expected gain (real option approach) than if disease increase is a logistic process.

## 5. Discussion

We have focused on the timing of a single application of treatment in a regional agricultural setting, over which there is risk of severe disease in some years but not necessarily in all. By allowing for uncertainty in the progression of the disease within a single season we were able to value the option to treat. Our results show that, even if the expected gain from immediate treatment is positive, there may still be value in waiting. The real option approach applied here identifies a delay strategy that maximizes the expected gain in any single season in the presence of uncertainty, while also exploring differences in disease increase. Whereas most theoretical analyses of real option approaches in natural systems assume exponential increase, many epidemics are intrinsically nonlinear, with density dependence limiting the availability of susceptible tissue. By using the logistic function as a simple exemplar of density-dependent increase in disease, our results show marked differences between the two models. In particular, we have shown that the qualitative response to the level of uncertainty in the path of infection of both the threshold value of treatment (*V*_*f*_) and the optimal time of treatment are highly dependent upon the model for disease increase (figure 2; see the electronic supplementary material, figure S2).

For systems in which the disease severity each season is highly predictable, the probability of exercising the option to treat over the whole season is high (see the electronic supplementary material, figure S3). Moreover, when it is never optimal to exercise the option to treat (*V* < *V*_*f*_ over the entire season), it was shown that the average gains from waiting under the real option approach and the gains under the cost–benefit approach are almost identical (figure 4*c*,*d*). The result holds for both disease models. It follows that for highly predictable epidemics, a decision-making agency would have more incentive to adopt a delay strategy (real option approach) than treating early under a cost–benefit approach. Such a choice would be based not only on the high probability of exercising treatment over the whole season, but also on the higher expected return secured by the real option approach (figure 4*e*,*f*).

For highly volatile systems, the probability of exercising the option to treat is very low (see the electronic supplementary material, figure S3). If a decision to delay treatment is taken, the large degree of variability in the severity of the epidemic each season may mean that the threshold value *V*_*f*_ that triggers treatment may not be attained. In this case, there may be an economic gain in treating earlier according to a cost–benefit approach but importantly this cannot be known in advance. However, when *V* > *V*_*f*_ is satisfied, a very high benefit occurs from delayed treatment (figure 4*e*,*f*).

The problem confronting a decision-making agency in controlling epidemics under uncertainty is whether to treat early, on the assumption that an epidemic might occur, or to delay the release of treatment until more is known about the likely progression of the epidemic. The solution depends upon the agency taking account of risk. Rather than a firm decision not to treat throughout the whole season, a decision-making agency may instead recommend continuous monitoring of the level of disease incidence against the threshold level required for immediate treatment. It is important to note that the threshold value *V*_*f*_ required for immediate treatment changes over time.

Further, our analyses show that the threshold value of treatment (*V*_*f*_) varies significantly with the level of uncertainty in the path of infection (*σ*) and the epidemiological model used to characterize the progression of disease. One of the main factors influencing the qualitative behaviour of *V*_*f*_, with respect to the level of uncertainty *σ*, is the relative duration of the cropping season with respect to the transmission rate of the pathogen. For short cropping seasons, the threshold value of treatment increases with the level of uncertainty in the path of infection, regardless of the epidemiological model. This result is consistent with common financial options, in which the exponential function is routinely used. However, for longer seasons, the density-dependent model, appropriate for many epidemics, leads to a concavity in the response of *V*_*f*_ to *σ* (figure 2). Whether or not a season is defined as long, depends upon the transmission rate (*β*) *inter alia* with a long season being one in which there is a high probability of disease extinction under a logistic stochastic process (see the electronic supplementary material).

The model presented here is an initial step towards understanding how irreversibility and flexibility affect optimal treatment decisions in agriculture under uncertainty, for which we have introduced a density-dependent model for disease increase. Further work is required to incorporate additional realism to the system. For instance, cryptic infection (in which infected individuals may transmit infection without exhibiting symptoms) is assumed to have very little or no effect on this model. This contrasts with many pathogens (Gilligan *et al.* 2007) in which there is a delay between infection and symptom expression. The model could also be extended from a single to multiple seasons by establishing how the initial inoculum for one season depends upon the epidemic dynamics in the previous season (Gilligan *et al.* 2007). Several other adaptations of the model may be introduced to increase realism in the decision-making process. For example, the approach may be adapted to introduce a decision-maker's risk preference (e.g. risk aversion). This may be done by modifying the objective function (utility function) so as to account for the effect of the decision-makers' risk attitude on the valuation of treatment, when the path of disease progression is uncertain. Other adaptation may replace a central agency that dictates the strategy for the entire landscape with locally informed but spatially coupled decisions that take account of heterogeneities in the landscape (Holt *et al.* 2006). Further work may also be undertaken to change the criterion for decision-making in order to distinguish public (as assumed above) from private benefits and costs of disease control (Perrings *et al.* 2005).

**Table 1.** Summary of variables and parameters.

symbol	definition
*variables*	
*I*(*t*)	the level of infection at time *t*
*V*(*I, t*)	the value of treatment at time *t*
*F*(*V*)	the value of the option to treat
*V*_*f*_(*t*)	the threshold value for immediate treatment at time *t*
*parameters*	
*p*	the monetary gain in yield per unit of averted infection
*D*	the costs of treatment
*r*	the discount rate
*β*	the transmission rate of infection
*σ*	the level of uncertainty in the path of infection
*I*_max_	the environmental carrying capacity
